# Quality of care in the EsSalud emergency service, northern Region, Peru

**DOI:** 10.25122/jml-2021-0254

**Published:** 2022-12

**Authors:** Delia Florencia Dávila Vigil, Carlos Alberto Chirinos Ríos

**Affiliations:** 1 School of Medicine, Señor of Sipan University, Chiclayo, Peru

**Keywords:** quality, user service, emergency service, user perception, user expectation, EsSalud

## Abstract

This study explores the user's quality of medical attention. The aim was to analyze the quality-of-care indicators in the EsSalud emergency service of the hospitals of the Lambayeque Juan Aita Valle Healthcare Network, according to the perceptions and expectations of the patients. The research has a descriptive, correlational, cross-sectional design, which includes the description, registration, analysis, and interpretation of the current nature of the quality of emergency medical care in the selected hospital institutions. The population is represented by patients who attended the emergency department of the hospital institution's understudy for one month. The sample was obtained using a simple random system. The technique used was the application of Servqual Quality of Emergency Care questionnaire. The instrument consisted of three parts, General Data, Expectations of the External User, and Perceptions of the External User. All statistical tests were processed using SPSS v.25 through the T-test of means. Higher customer dissatisfaction was revealed with regard to intangibility, lack of security, lack of reliability, and lack of empathy towards the user. User expectations and perceptions regarding the health service quality are also marked by dissatisfaction and great dissatisfaction.

## INTRODUCTION

Public policy on the quality of health progressively increases. Its most excellent development occurs mainly in the US and European countries [1], with user satisfaction being an indicator for the evaluation of the interventions of medical services, whose purpose is to obtain excellence in health services [2]. The quality of health services is a challenge for the health sector as it is based on improving institutional capacities for care and prioritizing people's lives [3].

Previous studies related to the problem in question highlight that patients feel satisfied when treated with respect, in hygienic environments, and when medical personnel is willing to listen to them with kindness [4]. This leads to high levels of satisfaction and preference for health institutions [5]. Among the main dimensions that can condition the perception of quality in care are the reliability and the capacity for rapid responses of the service [6], as well as empathy, technical equipment, infrastructure, competent staff, and the knowledge of the administrative and assistance personnel [7]. In addition, a study on the perception of the quality of care found that only 12.27% of the participants described the care in the health centers where it was performed as "good", meaning that the quality was unsatisfactory. Therefore, there is a need for the services to be improved [8]. Health must be conceived as a dimension of the quality of life, hence, a fundamental condition of human development. In this sense, health is a fundamental and inalienable right to which every person must have access, as enshrined in the International Covenant on Economic, Social, and Cultural Rights, recognizing that the right to health encompasses four essential and interrelated elements: availability, accessibility, acceptability, and quality.

Based on the above-stated information, the approach of strategic management appears, representing a qualitative leap for the management of health services since it allows them to recognize the person as a fundamental reference in the modern organization, be it user, customer, supplier, ally, competitor, regulator, or others. Strategic thinking forced the organizations to "reinvent" "from otherness" in what was called the "outside-in" strategy. The quality approach advances in specifying who is that "other", their needs, interests, motivations, and expectations, and how diverse, complex, and fickle it is. Despite this, this "other" is the center of the mission of the health organization. Therefore, it may be more appropriate to refer to Strategic Management in regard to Quality.

The development and strengthening of health services under the quality strategy indicates that it is the objective and tangible experience that puts providers and users in direct contact in implementing care processes. The quality approach seeks to strengthen the services that make up the care processes where the technical, interpersonal, and environmental dimensions are inextricably linked; through effective, ethical, and humane performance, proper use of technology, and other necessary resources.

In 1981 the World Health Organization (WHO) defined the concept of quality, referring to health services, as "the situation in which the patient is correctly diagnosed and treated according to current knowledge of science" [9]. Satisfaction is the feeling of pleasure or disappointment that results from comparing the performance or a perceived result of a product or person with the expectations [10]. Therefore, patient satisfaction results from the difference between the perception of the service received and the satisfaction of the expectations. If the expectations are higher than the actual quality of the service, satisfaction will decrease. These expectations are forged from their previous experiences, the socio-cultural context, and the commitments that the current health systems form in patients [11]. There is a direct relationship between perception and motivation in patients. How the motivated person acts depends on their perception of the situation. Perception is how an individual selects, organizes, and interprets the information received to create an intelligible image of the world [10].

In Latin America, there are still few national proposals for public health policies that comprehensively take on the challenge of quality. One of the most widespread experiences is the Mexican one, which foresaw the dignified treatment of both users of health services and their families in its health policy. Another relevant experience in Latin America is the Colombian one, which materialized by creating a Mandatory Quality Assurance System, organized in close correspondence with the challenges arising in the reform of the health system in that country, aimed at achieving universal insurance [12, 13].

In Peru, the Social Security (EsSalud) defines the quality of healthcare as the provision of services to individual and collective users in an accessible and equitable way through an optimal professional level, taking into account the balance between benefits, risks, and costs [14]. The National Quality Policy, directed by the Ministry of Health (MINSA), aims to improve the quality of health care in health service provider organizations through the implementation of the guidelines issued by the National Health Authority, which is responsible for quality management in health. Currently, there are signs of growing concern about the quality of care provided, where there are few strategies aimed at guiding the development of quality in a comprehensive, practical, and sustainable way, forcing the formulation of public policies that guide the behavior of health organizations toward improving the quality of care [15].

The National Health Policy Guidelines (2002) enhanced the development of the Concerted National Health Plan approved in 2007, which includes the concern for the development of quality of health care in the Fifth Guideline: Progressive improvement of access to quality health services, with the strategic objective of expanding the offer, improving the quality and organization of the health services in the sector according to the needs and demand of the users; defining, in addition, a set of goals for 2011. This orientation displays the Fifth Policy Guideline, specifying content and responsibilities in its implementation [16]. Notwithstanding the efforts made by MINSA and other Peruvian organizations, the level of quality has not been raised. The population perceives the low quality of care and appreciates a low level of quality and a worsening trend in the MINSA and EsSalud hospitals [17].

To deal with these problems, EsSalud framed the four significant institutional policies approved by General Management Resolution 771-GG-ESSALUD-2007 and its Directive 006-GG-ESSALUD-2007, promoting a set of initiatives to develop a culture of attention to the quality ensured. A Service Quality Program is considered, which includes a service policy or philosophy that responds to the institution's mission where it is established how the relationship and treatment of the insured should be. To implement this proposal, it was necessary to have management and measurement tools that ensure its execution, continuous improvement, and sustainability over time.

The Juan Aita Valle Healthcare Network of Lambayeque-Peru (RAL JAV, for its acronym in Spanish) EsSalud covers the entire Lambayeque region in Peru and small sectors of the La Libertad, Peru, and Cajamarca, Peru regions, away from their departmental capitals. RAL JAV has 25 establishments (all belonging to EsSalud) distributed as follows: 1 national hospital, two complexity level II hospitals, three complexity level I hospitals, four primary care centers III (CAP III) or polyclinics of increasing complexity, nine primary care centers II (CAP II) or medical centers, and six primary care centers (CAP I) or medical establishments and medical centers. According to their domicile geographic location, the population assigned to RAL JAV is 582,913 insured people who are cared for in the mentioned centers [18]. Based on the number of insured people in the JAV RAL in the last decade, 2008–2017, the population trend is projected under the previous behavior. In this sense, for the next ten years (2018–2027), overall population growth is projected, for which the current hospital infrastructure would be insufficient [18].

Since 2016, the Central Management of Attention to the Insured of the Social Security of Peru has been informing that the RAL JAV occupies the second place of the Networks of provinces with the highest number of requests for intervention: claims, petitions, queries, and suggestions for systematic attention [19]. It is also stated that only in 2016 were there 6,507 dissatisfactions; from January to October 2017, there were 5,086 dissatisfactions, with the following fundamental causes: delay in making an appointment, delay in attention, lack of medicine and pharmacy materials [19]. Likewise, in its report on the management of care requests for the Lambayeque Assistance Network, the comparative analysis from January to April 2017 and 2018 shows an increase in registrations in January (48%), February (47%), and April (11%), with an average increase in the registration of care requests of 18%. Also, in 2018 there was an increase in claims for care outside the care period from 1% in 2017 to 9% in 2018 [19]. This led to the RAL in the National Dissatisfaction Ranking for 2018. JAV is located in fifth place at the level of EsSalud establishments [19]. It was also found that emergency care in the researched hospitals was not following what was established. This is a current problem that is addressed in this research. From this problem, a proposal will be derived to improve the quality of care in hospital emergencies of the Lambayeque network [19].

Considering the problematic manifestations mentioned, it is perceived that in EsSalud, objective and subjective dissatisfactions persist, which limits the quality of care in the emergency service in the northern region of Peru. This study offers actual data on healthcare quality, according to the perception of emergency patients from the six hospitals in the Lambayeque EsSalud network. These data will be used to implement administrative and managerial actions to improve health services' attention with quality and efficiency. In this sense, decision-makers will have a set of dissatisfactions that must result from objective, consensual and incremental planning that aims to impact the quality of the health service significantly and positively and, therefore, the happiness of the people.

Given this, the research question is proposed: How does the Emergency Service of the RAL JAV hospitals belonging to EsSalud impact the quality of patient care? This study aims to analyze the indicators of quality of care in the EsSalud emergency service of the RAL JAV hospitals according to the perceptions and expectations of the patients.

## MATERIAL AND METHODS

The research presents a descriptive, correlational, cross-sectional design [20] that includes the description, registration, analysis, and interpretation of the current nature of the quality of emergency medical care in the hospital institutions under study. Likewise, it checks how the variables are interrelated to explain user satisfaction in the emergency service of the health institutions where the study was developed. In the same way, it determines the degree to which the variations in expectation are concomitant with the variation in the perception of satisfaction of hospital users. It is cross-sectional because the evaluation instrument is applied to patients who came for emergency care from the hospitals mentioned above in a finite timeframe. The design of this report follows the form: M→O, where M=Sample; O=Observation [21].

The population consisted of patients who attended the emergency department of the hospital for a month. To calculate the sample size for data collection, the statistical formula of finite populations was used. The sample was obtained using a simple random system. The formula used was the following:


$$N=\frac{z_{2}pqN}{e_{2}(N-1)+z_{2}pq}$$

Where n=Sample size; p=Proportion of external users who expect to be dissatisfied: 0.5; q=Proportion of external users who expect to be satisfied. Its value is (1 - p): 0.5; e=Standard error of 0.05 (for categories II and III of the establishment); z=1.96 (95% confidence interval); N=population of external users who attended in the last year or semester in the emergency services. The following inclusion criteria determined the sample: patients of both sexes, older than 18 years of age at the time they attend emergency health care at the six RAL JAV EsSalud Hospitals, and consent to be surveyed. Exclusion criteria: patients with physical or mental disabilities who do not answer all of the questions. Systematic sampling was implemented based on the following criteria: To apply the survey systematically, the number of users who attended in the last week was divided by the size of the sample obtained, which established how many users should be interviewed: (677×7)/383=12.

The Servqual Quality of Emergency Care questionnaire was applied [22], structured by 22 questions related to the infrastructure, personnel, and emergency equipment of the hospitals, as mentioned above. The data collection instrument consisted of 3 parts. The first part included the general data, respectively age, sex, level of study, and type of patient insurance. The second part mentioned the expectations of the external user consisting of 22 questions divided into five dimensions, each question with seven alternatives according to the Likert scale. The dimensions were tangibility, reliability, prompt response, safety projection, and empathy. Perceptions of the external user consisted of 22 questions. Each one was divided into five dimensions: tangibility, reliability, prompt response, safety projection, and empathy. For the response alternatives (in expectations and perceptions), a Likert scale was used. Values were established for each question and corresponded to expectations and perceptions, whose difference is between +7 and -7. These results were called Gaps according to the operational definition of the instrument.

The Likert-type tests' data were transformed into quantitative data with values 1 to 7, which assume their corresponding weight. All statistical tests were processed with the statistical package SPSS *vs*. 17. The level of significance used was p=0.05. Statistical processing of all the data obtained was carried out. Finally, the analysis was performed for the quality of emergency care at the H-NAMP, H-1CPN, H-HSEN, H-IABN, H-IIJEN, and H-NAAA hospitals (Table 1).

**Table 1:** List of social security hospitals (EsSalud) included in the study.

N°	Hospital name	Coding
**1**	Naylamp Hospital, Lambayeque-Peru	H-NAMP
**2**	Hospital I, Chepén, La Libertad, Peru	H-1CPN
**3**	Hospital II, Luis Heysen Inchásticategui, Lambayeque-Peru	H-HSEN
**4**	Hospital I, Agustín Arbulú Neyra, Lambayeque-Peru	H-IABN
**5**	Hospital II, Jaén, Cajamarca-Peru	H-IIJEN
**6**	Almanzor Aguinaga Asenjo National Hospital, Lambayeque-Peru	H-NAAA

According to the perception of the external user, the statistical analysis was carried out by analysis of variance. The analysis of the intervening variables will be carried out through the T-test of means. The level of significance used was p=0.05.

## RESULTS

In Table 2, it is highlighted that the emergency service patients in the social security hospitals EsSalud are dissatisfied in 55.81% of the cases, with great dissatisfaction in 17.83% of the cases. In total, 73.64% of patients show dissatisfaction.

**Table 2:** Quality of care of the emergency service according to the indicators of perceptions and expectations of the patients.

Attention quality	H-NAMP		H-1CPN		H-HSEN		H-IABN		H-IIJEN		H-NAAA		Total	
	n	%	n	%	n	%	n	%	n	%	n	%	n	%
**Expectation exceeded**	4	5.06	1	3.33	28	22.58	10	25.64	6	15.38	34	44.74	83	21.45
**Expectation satisfied**	6	7.59	0	0.00	11	8.87	2	5.13	0	0.00	0	0.00	19	4.91
**Dissatisfaction**	55	69.62	15	50.00	76	61.29	20	51.28	26	66.67	24	31.58	216	55.81
**Great dissatisfaction**	14	17.72	14	46.67	9	7.26	7	17.95	7	17.95	18	23.68	69	17.83
**Total**	79	100	30	100	124	100	39	100	39	100	76	100	387	100

In Table 3, the quality of care of the emergency service in the RAL JAV Hospitals according to the indicators of perceptions and expectations of the patients based on the indicators: reliability, response capacity, safety, empathy, and tangibility. In terms of reliability, there is dissatisfaction in 47.3% of the cases and great dissatisfaction in 16% of the cases (63.3% in total). Likewise, when considering the response capacity, there is dissatisfaction in 39.8% of the cases and great dissatisfaction in 20.9% of the cases (60.7% in total). Regarding safety, they present dissatisfaction in 48.8% of instances and great dissatisfaction in 15.8% of instances (64.6% in total). Regarding empathy, dissatisfaction is found in 43.2% of the cases and great dissatisfaction in 16.5% of the cases, adding up to 59.7%. Moreover, tangibility dissatisfaction is found in 45.7% of cases and great dissatisfaction in 20.9% of cases (66.6% in total).

**Table 3:** Quality of emergency service care according to the indicators of patient perceptions and expectations.

	H-NAMP		H-1CPN		H-HSEN		H-IABN		H-IIJEN		H-NAAA		Total	
	n	%	n	%	n	%	n	%	n	%	N	%	n	%
**Reliability**														
Expectation Exceeded	6	1.6	1	0.3	32	8.3	9	2.3	5	1.3	32	8.3	85	22.0
Satisfied Expectation	17	4.4	0.0	0.0	23	5.9	10	2.6	5	1.3	2	0.5	57	14.7
Dissatisfaction	42	10.9	16	4.1	62	16.0	13	3.4	21	5.4	29	7.5	183	47.3
Great dissatisfaction	14	3.6	13	3.4	7	1.8	7	1.8	8	2.1	13	3.4	62	16.0
**Answer's capacity**														
Expectation Exceeded	7	1.8	2	0.5	33	8.5	9	2.3	3	0.8	32	8.3	86	22.2
Satisfied Expectation	22	5.7	1	0.3	27	7.0	4	1.0	6	1.6	6	1.6	66	17.1
Dissatisfaction	38	9.8	11	2.8	57	14.7	9	2.3	19	4.9	20	5.2	154	39.8
Great dissatisfaction	12	3.1	16	4.1	7	1.8	17	4.4	11	2.8	18	4.7	81	20.9
**Safety**														
Expectation Exceeded	6	1.6	0.0	0.0	20	5.2	10	2.6	3	0.8	35	9.0	74	19.1
Satisfied Expectation	18	4.7	0.0	0.0	28	7.2	9	2.3	6	1.6	2	0.5	63	16.3
Dissatisfaction	39	10.1	16	4.1	71	18.3	15	3.9	25	6.5	23	5.9	189	48.8
Great dissatisfaction	16	4.1	14	3.6	5	1.3	5	1.3	5	1.3	16	4.1	61	15.8
**Empathy**														
Expectation Exceeded	7	1.8	1	0.3	27	7.0	9	2.3	4	1.0	36	9.3	84	21.7
Satisfied Expectation	25	6.5	2	0.5	25	6.5	10	2.6	8	2.1	2	0.5	72	18.6
Dissatisfaction	37	9.6	12	3.1	63	16.3	13	3.4	21	5.4	21	5.4	167	43.2
Great dissatisfaction	10	2.6	15	3.9	9	2.3	7	1.8	6	1.6	17	4.4	64	16.5
**Tangibility**														
Expectation Exceeded	1	0.3	2	0.5	25	6.5	8	2.1	4	1.0	36	9.3	76	19.6
Satisfied Expectation	18	4.7	1	0.3	24	6.2	5	1.3	3	0.8	1	0.3	52	13.4
Dissatisfaction	41	10.6	13	3.4	66	17.1	13	3.4	26	6.7	19	4.9	178	45.7
Great dissatisfaction	19	4.9	14	3.6	9	2.3	13	3.4	6	1.6	20	5.2	81	20.9

The patients treated in the emergency service of the Hospitals were mainly female, 68.73% compared to 31.27% of male patients. These findings of a broad age range coincide with those provided by Huarcaya H, 2015 in Andahuaylas-Peru, where it was found that 76.21% of the users of the emergency service were women and 23.79% were men [8].

The patients treated in the emergency service of the hospitals had an average of 40.40 years in the female sex and 48.83 years in the male sex, making the notation that a high standard deviation was obtained for which the data is extended over a broader range of values. These findings of a broad age range are also consistent with what was found by Huarcaya H, 2015 in Andahuaylas-Peru, where 64.31% of the emergency service users were between 20 and 40 years old and 30.48% between 41 and 60 years [8].

In Table 4, the patients treated in the emergency service of the hospitals by the type of insurance were primarily the Compulsory Insured (70.80%) followed in frequency by the Right Holder with 15.50%, and the type of insured who attended less frequently was the Pensioners with 5.17%.

**Table 4:** Sociodemographic characteristics of the users and the insurance type of the patient.

Type of social security	Male		Feminine		Total	
	n	%	n	%	n	%
**Obligatory**	98	25.32	176	45.48	274	70.80
**Right Holder**	10	2.58	50	12.92	60	15.50
**Optional**	8	2.07	25	6.46	33	8.53
**Pensioner**	5	1.29	15	3.88	20	5.17
**Total**	121	31.27	266	68.73	387	100.00

### Ethical considerations

The research assumed two fundamental ethical principles: (1) Informed Consent; (2) Confidentiality and Anonymity [23]. Moreover, it takes into account the principles of the Belmont Report, which includes three principles that have been put into practice in educational research: The principle of Respect for Human Dignity, the Principle of Justice, and the Principle of Beneficence [24].

## DISCUSSION

This result does not agree with a study carried out in Spain, where the satisfaction percentage was 84.7% [4]. It also does not agree with the findings of a study from Cuba, where satisfaction situations above 90% were observed in indicators such as Willingness to attend again, Satisfaction with the quality of care, and willingness to recommend the service to other patients [5]. However, their results are similar to those found in El Salvador, where dissatisfaction was found in the emergency of Social Security Hospital in the process variables (Technical and Human Quality) and the efficiency variable [25]. They also agree with the results obtained in a study developed in Lima-Peru, where a high degree of dissatisfaction (90.1%) was found on the part of the patients [6]. A similar situation was found in Hospital I Santa Margarita, Andahuaylas-Peru; There, in the emergency service, the care was found unsatisfactory [8].

Due to those mentioned above, it was found that tangibility obtained 66.6%, security 64.6%, reliability 63.3%, response capacity 60.7%, and empathy obtained 59.7%. These results were contrary to those found in the Sergio E. Bernales Hospital, Lima-Peru, where the dimension with the most significant dissatisfaction was the response capacity, reaching 92.54% [6]. In Tacna, the most significant dissatisfaction was with reliability (76.58% of cases) [7].

These data cannot be contrasted with previous studies since no detailed bibliography was found on the type of insured treated in emergencies at EsSalud hospitals in Peru.

In Table 5 regarding the expectations and perceptions of emergency patients related to the quality-of-service care and its association with sex in the hospitals of the RAL JAV EsSalud, it was found that dissatisfaction and great dissatisfaction (55.81% and 17.83%, respectively) added up to 73.64% with a predominance in the female sex, reaching a dissatisfaction and great dissatisfaction of 73.64%. When the ANOVA test on the correlation between sex and quality of care was applied, a p-value of 0.0774 greater than 0.05 was obtained, the maximum level allowed; therefore, no statistical relationship was found between sex and quality of care. When considering its association with age, it was found that dissatisfaction and great dissatisfaction (55.81% and 17.83%, respectively) amounted to 73.64%, predominantly in the age group of 31 to 60 years, reaching a dissatisfaction and great dissatisfaction of 41.6%. There was a statistically significant difference between age and quality of care (p-value=0.0154). Regarding the relationship between satisfaction and the type of social security, dissatisfaction was 73.64%, being the most frequent patient with the type of compulsory insurance. No statistically significant relationship was found between the age of the patients and the level of satisfaction.

**Table 5:** Characterization of patients and patient satisfaction of social security hospitals.

Quality of health care	Feminine		31–60 years		Obligatory	
	n	%	n	%	n	%
**Expectation exceeded**	52	13.44	40	10.34	64	16.54
**Expectation satisfied**	9	2.33	7	1.81	15	3.88
**Dissatisfaction**	155	40.05	120	31.01	148	38.24
**Great dissatisfaction**	50	12.92	41	10.59	47	12.14
**Grand total**	266	68.73	208	53.75	274	70.80
**Reliability index**	p=0.0774	-	p=0.0154	-	p=0.0055	-

## CONCLUSION

Most patients were dissatisfied (55.81%) and greatly dissatisfied (17.82%) with the quality of care in the emergency service in the hospitals included in the study. The dimensions most frequently representative of dissatisfaction in service quality were tangibility, lack of security, lack of reliability, and lack of empathy towards the user.

The expectations and perceptions of emergency patients related to quality-of-service care have a statistical association with gender since men have higher expectations than women (25.62% and 19.55%, respectively). Dissatisfaction in males is 50.41%, and in females, 58.27%, with no statistically significant relationship. Dissatisfaction was high in all age groups, respectively, patients under 31 years old (46.08%), patients between 31 and 60 years old (52.63%), and those over 60 years old (34.61%), with a statistically significant relationship.

The expectations and perceptions of emergency patients related to the quality of emergency service care according to the type of insured were marked by dissatisfaction and great dissatisfaction (55.81% and 17.83%, respectively), with the mandatory insurance group being predominant, reaching a dissatisfaction and great dissatisfaction of 50.38%, with a statistically significant relationship.

The proposal to improve processes in emergency services care is related to user orientation, improving user care, and improving the resolution capacity of emergency services.

This research shows the need for objective proposals to improve the quality of care in the emergency service in the hospitals mentioned above. Proposals should be aimed at sensitizing and training the admission administrative staff of the emergency service to provide adequate information and guidance and train the health team on social skills for the humane treatment necessary to safeguard the insured rights to quality care. Likewise, improve the management capacity of representatives of the Social Security of EsSalud, nationally, regionally, and locally, to upgrade the technological equipment, supplies, and necessary medicines.

These findings also demand the need for periodic systematic evaluation of user satisfaction and subsequent analysis of the results by the RAL JAV management team and each hospital management team that makes up the RAL JAV.

## Data Availability

Further data is available from the corresponding author on reasonable request.
